# Interleukin-17A directly triggers Epstein-Barr virus lytic reactivation in latency I human B-cell models

**DOI:** 10.1128/jvi.00813-26

**Published:** 2026-09-24

**Authors:** Janardhan Avilala, Hirotomo Dochi, Ramsy Abdelghani, David Becnel, Grace Maresh, Xin Zhang, Li Li, Mark Sides, Gilbert Morris, Hong Liu, Zhen Lin

**Affiliations:** 1 Department of Pathology and Laboratory Medicine, Tulane University School of Medicine 12255, New Orleans, Louisiana, USA; 2 Tulane Cancer Center, Tulane University School of Medicine 12255, New Orleans, Louisiana, USA; 3 Section of Pulmonary and Critical Care Medicine and Interventional Pulmonology, Department of Medicine, Tulane University School of Medicine 12255, New Orleans, Louisiana, USA; 4 Translational Cancer Research Laboratory, Ochsner Clinic Foundation 633467 , New Orleans, Louisiana, USA; 5 Section of Hematology and Medical Oncology, Department of Medicine, Tulane University School of Medicine 12255, New Orleans, Louisiana, USA; 6 Department of Biochemistry and Molecular Biology, Tulane University School of Medicine 12255, New Orleans, Louisiana, USA; University of Toronto, Toronto, Ontario, Canada

**Keywords:** Epstein-Barr virus, EBV, interleukin-17A, IL-17A, lytic reactivation, cytokine signaling, NF-κB, MAPK, RNA-seq

## Abstract

Epstein-Barr virus (EBV) is a ubiquitous human gammaherpesvirus implicated in inflammatory, malignant, and autoimmune diseases. Although EBV toggles between latent and lytic states, the host cues controlling this switch remain incompletely defined. Interleukin-17A (IL-17A), a signature Th17 cytokine, is enriched in EBV-associated inflammatory settings, and EBV products can augment IL-17A responses. Whether IL-17A directly regulates the EBV life cycle has remained unknown. Here, we show that IL-17A alone is sufficient to induce EBV lytic reactivation in latency I human B-cell models. IL-17A increased BZLF1/Zta, activated the BZLF1 promoter (Zp), induced early and late lytic genes and proteins, and promoted DNase-resistant EBV DNA release and infectious virion production. Mechanistically, IL-17A activated NF-κB and ERK/p38 MAPK signaling, including phosphorylation of p65, ERK1/2, and p38. IL-17RA blockade reduced IL-17A-induced signaling and lytic reactivation, and pharmacologic inhibition of NF-κB or p38 MAPK signaling attenuated EBV reactivation. Among IL-17 family members tested, only IL-17A induced reactivation. RNA-seq provided a transcriptome-wide view of the viral and cellular response to IL-17A, revealing broad EBV lytic transcription and inflammatory, NF-κB-associated, and B-cell activation programs. IL-17A responsiveness was latency- and cell-state restricted: latency I B-cell models responded, whereas latency II/III, lymphoblastoid, and EBV-positive epithelial models did not. Subinducing IL-17A (1 ng/mL) was insufficient alone but enhanced B-cell receptor-mediated reactivation, suggesting threshold-lowering activity in inflammatory B-cell niches. Together, these findings identify IL-17A as a receptor-linked inflammatory cue for EBV lytic entry and provide the first demonstration that IL-17A alone directly triggers EBV lytic reactivation in human cells.

**IMPORTANCE:**

Epstein-Barr virus (EBV) persists for life by maintaining latency with lytic reactivation, yet the inflammatory cues that initiate this switch remain poorly defined. Here, we identify interleukin-17A (IL-17A), a Th17 cytokine, as a direct trigger of EBV lytic reactivation in latency I human B-cell models. IL-17A activates IL-17RA-linked NF-κB/MAPK signaling, induces BZLF1/Zta and downstream lytic genes, and promotes DNase-resistant EBV DNA release and infectious virion production. IL-17RA blockade and inhibition of NF-κB/p38 MAPK signaling reduce reactivation, supporting a receptor-linked mechanism rather than nonspecific stress. Subinducing IL-17A (1 ng/mL) was insufficient to reactivate EBV alone but enhanced B-cell receptor-mediated reactivation, suggesting threshold-lowering activity in inflammatory B-cell niches. The response is restricted by latency and cell state, indicating that IL-17A signaling must be coupled to a permissive EBV lytic switch. These findings connect Th17-skewed inflammation to EBV reactivation burden and suggest that IL-17A/IL-17R signaling may influence EBV pathogenesis in inflammatory and malignant settings.

## INTRODUCTION

Epstein-Barr virus (EBV) is a ubiquitous human γ-herpesvirus that establishes lifelong infection and contributes to a spectrum of inflammatory and malignant diseases (1–8). A defining feature of EBV biology is its ability to toggle between latency and lytic replication. Entry into the lytic cycle hinges on the induction of the viral immediate-early transactivators BZLF1 (Zta) and BRLF1 (Rta), which launch the downstream lytic transcriptional cascade and enable production of progeny virions (9–11). Understanding what cellular cues flip this latency/lytic switch remains central to explaining EBV pathogenesis in tissues where inflammation, hypoxia, or other stressors are prominent (12, 13).

Multiple host pathways can precipitate EBV reactivation *in vitro* and *in vivo* (13–17). Canonical inducers include B-cell receptor (BCR)-driven signaling and cellular stress responses that converge on PKC, MAPK (ERK, JNK, and p38), PI3K, and ATM networks to activate the BZLF1 promoter (Zp). Additional physiologic triggers have been described. Hypoxia/HIF-1α can bind Zp to drive BZLF1. Inflammatory eicosanoids such as COX-2/PGE2 promote reactivation via EP receptor signaling. TGF-β induces lytic entry in epithelial and other EBV-infected cells. Collectively, these studies establish that EBV is responsive to host stress and inflammatory cues. However, the role of cytokines as direct lytic triggers is far less defined. Recent work further showed that germinal center cytokines can remodel EBV latency gene expression through JAK/STAT-dependent epigenetic regulation, underscoring the ability of host cytokine environments to shape EBV transcriptional programs (18).

Interleukin-17A (IL-17A), often referred to simply as IL-17, is the prototypical Th17 cytokine and has potent context-dependent effects on stromal and epithelial cells (19, 20). In this study, recombinant human IL-17A was the primary cytokine tested and is referred to as IL-17 unless otherwise indicated. Other IL-17 family members, including IL-17D and IL-17F, are specified explicitly. IL-17A signals through heterodimeric receptors that typically include IL-17RA and IL-17RC, recruiting the adaptor ACT1/TRAF3IP2 and TRAF proteins to activate NF-κB, AP-1/MAPK, and post-transcriptional programs that stabilize target mRNAs. These features allow IL-17A to reshape tissue responses and, in some settings, to intersect with oncogenic and stress-response pathways that could, in principle, influence EBV’s lytic switch (19, 20).

IL-17/Th17-skewed inflammation is detected across several EBV-associated contexts (21–23). In nasopharyngeal carcinoma (NPC), a tumor tightly linked to EBV, tumor tissues are enriched for Th17 cells, and IL-17 can promote NPC cell proliferation *in vitro*, underscoring the functional relevance of this axis in EBV-associated microenvironments. Conversely, classical Hodgkin lymphoma shows nuanced patterns where Th17 engagement can be stronger in EBV-negative disease, highlighting disease-specific heterogeneity. These observations nevertheless situate IL-17 as a plausible modulator in EBV-related tissues (21–23).

Notably, the directionality most often documented in the literature flows from EBV to IL-17 (24–26). That is, EBV nucleic acids and proteins augment IL-17 production and/or Th17 polarization through endosomal TLR pathways or other innate sensors. For example, purified EBV DNA enhances IL-17 via TLR-dependent mechanisms in mice, and the EBV dUTPase (a pathogen-associated molecular pattern) can elicit Th17-type cytokines. These findings imply that EBV reactivation or antigen release could fuel IL-17-rich milieus, but whether IL-17, in turn, directly regulates the EBV life cycle has remained an open question.

Cross-species precedent suggests that such a link is plausible. In a murine γ-herpesvirus model (MHV68), IL-17 signaling supports viral reactivation and *de novo* lytic infection, and genetic or pharmacologic disruption of IL-17 pathways attenuates the establishment of chronic infection (27). This work positions IL-17 as a pro-viral host cue in γ-herpesvirus biology, yet direct evidence in human EBV-infected cells has been lacking.

Here, we address this gap by testing whether IL-17A alone is sufficient to trigger EBV lytic reactivation in latently infected human B cells and by defining the signaling events that connect IL-17A receptor engagement to the EBV lytic switch. Using RT-qPCR, immunoblotting, Zp dual-luciferase reporter assays, flow-based lytic reporter quantification, DNase-resistant extracellular EBV DNA, Raji cell infectivity assay, and RNA-seq, we show that IL-17A induces BZLF1 and downstream early and late viral genes and drives productive lytic output, including DNase-resistant EBV DNA release and infectious virion production. We further show that IL-17A activates NF-κB and ERK/p38 MAPK signaling in latency I B cells and that IL-17RA blockade and inhibition of NF-κB or p38 MAPK signaling reduce IL-17A-driven reactivation. RNA-seq provides a transcriptome-wide view of the overall viral and cellular response to IL-17A, revealing broad EBV lytic transcription and accompanying inflammatory, NF-κB-linked, and B-cell activation programs. By testing additional latency II/III/LCL and epithelial models, we show that IL-17A responsiveness is latency- and cell-state restricted. Importantly, subinducing IL-17A (1 ng/mL) was insufficient to reactivate EBV alone but enhanced B-cell receptor-mediated reactivation, suggesting threshold-lowering activity in inflammatory B-cell niches. Together, these results identify IL-17A as an inflammatory cytokine cue that engages IL-17RA-linked NF-κB/MAPK signaling to drive EBV lytic entry in latency I-permissive B-cell contexts and lower the threshold for BCR-mediated reactivation at lower cytokine doses.

## MATERIALS AND METHODS

### Cell culture

The EBV-positive Akata cell line (type I latency) was established from an EBV-positive Burkitt’s lymphoma from a Japanese patient with a t(8:14) chromosome translocation (28). The EBV-positive Mutu (Mutu I) cell line (type I latency) was derived from an EBV-positive Burkitt’s lymphoma biopsy specimen from a Kenyan patient with a typical t(8:14) chromosome translocation (28). Rael is an EBV-positive Burkitt’s lymphoma cell line (type I latency). SNK6 is an EBV-positive NK/T-cell lymphoma-derived (NK-lineage) cell line (type II latency). Raji, Mutu III, JY, X50-7, and human PBMC-derived lymphoblastoid cell line are EBV-positive B-cell models with type III latency. C666-1 is an EBV-positive nasopharyngeal carcinoma cell line (type II latency), and SNU719 is an EBV-positive gastric carcinoma cell line (type II latency). DG75 is an EBV-negative Burkitt’s lymphoma cell line. 293 is an EBV-negative human embryonic kidney cell line.

Akata-BMRF1p-GFP and Mutu-BMRF1p-GFP cell lines were generated by stable maintenance of the pCEP4-BMRF1p-GFP reporter vector and were cultured under hygromycin B selection (50 µg/mL) (10). Akata-EBV-eGFP cells, which carry recombinant eGFP-expressing EBV, were maintained under G418 selection (800 µg/mL) and used for infectious virus production assays. All cell lines were maintained in either Dulbecco’s modified Eagle’s medium (DMEM; ThermoFisher Scientific, Cat# SH30243.02) for adherent cells or RPMI 1640 (ThermoFisher Scientific, Cat# SH30027.02) for suspension cells, supplemented with 10% fetal bovine serum (FBS; ThermoFisher Scientific, Cat# 10437-028), penicillin, streptomycin, and glutamine. Cells were grown at 37°C in a humidified 5% CO_2_-containing atmosphere.

### IL-17A treatment, neutralization, and IL-17 family stimulation

Recombinant human IL-17A (carrier-free; Peprotech, Cat# 200-17-100UG) was reconstituted in sterile PBS containing 0.1% BSA. For dose-response experiments, cells were treated with IL-17A at 0.1–1,000 ng/mL. Unless otherwise indicated, 500 ng/mL IL-17A was used to achieve maximal and reproducible induction. For neutralization assays, IL-17A was mixed with neutralizing anti-human IL-17A monoclonal antibody (R&D Systems, Cat# MAB31711-100) or a matched isotype control (Santa Cruz Biotechnology, Cat# SC-2025) and preincubated for 1 h at 37°C prior to addition to cells. For IL-17 family assays, cells were treated with IL-17A, IL-17D (MedChemExpress, Cat# HY-P700106AF), and IL-17F (MedChemExpress, Cat# HY-P70540) under matched conditions, and EBV reactivation was assessed as indicated.

### IL-17RA blockade

For IL-17RA blockade, cells were pretreated with neutralizing anti-human IL-17RA antibody (R&D Systems, Cat# AF177-SP) or matched isotype control (R&D Systems, Cat# AB-108-C) for 30 min at 37°C before stimulation with recombinant IL-17A. IL-17A was then added directly to the antibody-containing culture, and the antibody or isotype control was maintained in the medium throughout the subsequent treatment period until harvest. Early pathway blockade was assessed after 2 h of IL-17A stimulation by immunoblotting for phospho-p65, phospho-ERK1/2, and phospho-p38, with total p65, ERK1/2, and p38 used as pathway controls. EBV lytic reactivation was assessed after 24 h of IL-17A stimulation by RT-qPCR and immunoblotting as indicated.

### Pathway inhibitor treatment

To minimize nonspecific toxicity from prolonged inhibitor exposure, BMS-345541 and Adezmapimod were applied as transient early-signaling inhibitors. Cells were pretreated with BMS-345541 or Adezmapimod for 1 h before IL-17A stimulation. For phosphorylation assays, IL-17A and the indicated inhibitor were then co-applied for 2 h, and whole-cell lysates were collected immediately for immunoblotting. For Zta induction assays, cells were pretreated with inhibitor for 1 h and then co-treated with IL-17A plus inhibitor for 2 h. The inhibitor-containing medium was then replaced with fresh medium containing IL-17A or vehicle, and cells were harvested 16 h after IL-17A addition for immunoblotting of lytic proteins. Inhibitor-alone controls were processed in parallel without IL-17A.

### Low-dose IL-17A and BCR co-stimulation

To test whether subinducing IL-17A could lower the threshold for BCR-mediated EBV reactivation, cells were treated with subthreshold anti-Ig stimulation alone (0.2 μg/mL) or the combination of IL-17A (1 or 10 ng/mL) plus anti-Ig (0.2 μg/mL) for 24 h. EBV lytic induction was assessed by RT-qPCR.

### Transfection

Transient transfection experiments were performed by using either a modified version of the calcium phosphate precipitation procedure for adherent cells (29) or the Amaxa electroporation method for suspension cells (30). For the calcium phosphate precipitation procedure, 1 × 10^6^ cells were plated onto 100-mm-diameter tissue culture dishes. The following day, the medium was replaced with 8 mL of fresh supplemented DMEM; 4 h later, 0.5 mL of 1× HEPES-buffered saline (0.5% HEPES, 0.8% NaCl, 0.1% dextrose, 0.01% anhydrous Na_2_HPO_4_, and 0.37% KCl [pH 7.10]) was mixed with a total of 30 µg of plasmid DNA (effector plasmids were added in the amounts indicated in the figure legends, and carrier DNA [pUHD10] was added to make a total of 30 µg). A volume of 30 µL of 2.5 M CaCl_2_ was added, and samples were mixed immediately. Precipitates were allowed to form at room temperature for 20 min before being added dropwise to cells. Cells were incubated at 37°C with 5% CO_2_ for 16 h before the medium was replaced with 10 mL of fresh supplemented DMEM. For the Amaxa electroporation method, cells were placed in antibiotic-free RPMI medium 2 days before electroporation. For each transfection, 2 × 10^6^ cells in 100 μL of Nucleofector Solution R (Mirus, Cat# MIR50118) were electroporated with a total of 5 μg of plasmid DNA (containing both effector and carrier DNA [pUHD10]) and transferred to a 6-well plate with 1.5 mL of medium per well. After 24 h, 1.5 mL of fresh RPMI medium was added to each well. Cells were incubated for another 24 h before harvesting.

### Dual-luciferase reporter assay

Cells were transfected with a BZLF1 promoter (Zp)-driven firefly luciferase reporter together with a Renilla luciferase control plasmid. Following recovery, cells were stimulated with recombinant human IL-17A for 24 h. Firefly and Renilla activities were measured using a dual-luciferase reporter assay system (Promega, Cat# E1910), and Zp activity was expressed as Firefly/Renilla relative to vehicle control.

### Flow cytometry for BMRF1p-GFP reporter cells

Akata and Mutu I cells stably expressing GFP under the control of the BMRF1 promoter (i.e., Akata-BMRF1p-GFP and Mutu-BMRF1p-GFP) were treated with control vehicle, IL-17A (500 ng/mL), or anti-Ig (10 μg/mL) for 48 h and analyzed by flow cytometry. GFP-positive cells were quantified after exclusion of debris and dead cells, and the percentage of GFP-positive cells was used as a measure of early lytic activation.

### Ganciclovir treatment

Cells were treated with IL-17A in the presence or absence of ganciclovir (10 µg/mL; MedChemExpress, Cat# HY-13637). For the control experiment in Fig. 3A, RNA was harvested 24 h after IL-17A treatment, and EBV lytic transcripts were quantified by RT-qPCR. For the early timing-control experiment in Fig. S1, Akata and Mutu I cells were treated with vehicle, IL-17A, ganciclovir alone, or IL-17A plus ganciclovir for 4 h, and Zta/BZLF1 and BMRF1 transcripts were quantified by RT-qPCR.

### RNA preparation

Whole cell RNA preparations were carried out using TRIzol reagent (Thermo Fisher, Cat# 15596) according to the vendor’s recommended protocol.

### RT-qPCR analysis

cDNA was synthesized from total RNA using iScript gDNA Clear cDNA Synthesis Kit (Bio-Rad, Cat# 172-5035). qPCR analysis was performed using iQ SYBR Green Supermix (Bio-Rad, Cat# 170-8882) on a Bio-Rad CFX96 instrument as follows: 1 μL of cDNA and 1 μL of 10 μM primers were mixed with 10 μL of SYBR Green Supermix and 8 μL nuclease-free H_2_O to a 20 μL reaction volume. Polymerase was activated, and cDNA was denatured at 95°C for 5 min. cDNA was then amplified for 40 cycles with 15 s denaturation at 95°C, 60 s annealing/extension, and plate reading at 60°C. Melting curve analysis was performed at temperatures from 60°C to 90°C with 0.5°C increment per 5 s.

For treatment-induced gene-expression analyses, relative expression was calculated using the comparative ΔΔCt method. Data are displayed either as fold change (2^−ΔΔCt^) or as log2(fold change), as indicated on each *y*-axis. For baseline comparison of IL-17 receptor family and TRAF3IP2/ACT1 transcript abundance across cell lines (Fig. 6C), Ct values were normalized only to GAPDH to generate ΔCt values, where ΔCt = Ct(target gene) − Ct(GAPDH). Values were plotted as 2^−ΔCt^ without using a cell-line calibrator. Higher 2^−ΔCt^ values indicate higher transcript abundance relative to GAPDH. Samples with no detectable amplification were marked as not detected. Technical triplicates were averaged for each biological sample before calculating group means. Primer sequences are listed in Table S1.

### Western blot analysis

The cells were pelleted at the desired time point and washed with 1× PBS, and then the cells were immediately suspended in 100 μL of 1× Laemmli sample buffer and boiled for 15 min at 95°C to denature the genomic DNA. Protein concentrations of the whole-cell extracts were measured with the Bio-Rad protein assay kit according to the manufacturer’s instructions. Equal amounts of protein were subjected to SDS-polyacrylamide gel electrophoresis and transferred to PVDF membranes (Whatman). The blots were blocked for 60 min in Tris-buffered saline containing 5% nonfat powdered milk and 1% FBS and then incubated with the primary antibody (in blocking buffer) overnight at 4°C. The blots were washed three times with 1× TBST (140 mM NaCl, 3 mM KCl, 25 mM Tris-HCl [pH 7.4], and 0.1% Tween 20) (each wash was carried out for approximately 15 min). To detect the chemiluminescence signals, the blots were then incubated with horseradish peroxidase-conjugated secondary antibody (Bio-Rad) in blocking buffer for 1 h at room temperature. Blots were washed as described above and analyzed with an enhanced chemiluminescence detection system (Perkin-Elmer) according to the manufacturer’s recommendations. Signals were acquired using a Bio-Rad ChemiDoc imaging system or by exposure to Fuji Super RX films (Fujifilm), followed by film scanning. To detect infrared fluorescence signals, an Odyssey infrared imaging system (Li-Cor Biosciences) was used with a secondary antibody (IRDye 680RD goat anti-rabbit antibody; Li-Cor, Cat# 925-68071). Immunoblot figure panels were assembled from the original image files using uniform brightness/contrast or level adjustments applied to the entire blot image or panel. No selective enhancement, erasure, cloning, masking, background filling, or local manipulation was performed.

The following primary antibodies were used for Western blot analysis: anti-Zta MAb (Santa Cruz Biotechnology, Cat# sc-53904), anti-BMRF1 MAb (Santa Cruz Biotechnology, Cat# SC-58121), anti-VCA/p18 (ThermoFisher, Cat# MAB8184), anti-GAPDH (Santa Cruz Biotechnology, Cat# SC-47724), anti-phospho-NF-κB p65 (Ser536) (Cell Signaling Technology, Cat# 3033T), anti-NF-κB p65 (Santa Cruz Biotechnology, Cat# SC-8008), anti-phospho-ERK1/2 (Thr202/Tyr204) (Cell Signaling Technology, Cat# 4376T), anti-ERK1/2 (Santa Cruz Biotechnology, Cat# SC-514302), anti-phospho-p38 (Thr180/Tyr182) (Cell Signaling Technology, Cat# 4511T), and anti-p38 (Santa Cruz Biotechnology, Cat# SC-7972).

### Analysis of DNase-resistant EBV DNA

Cell-free, virion-associated EBV DNA was quantified from culture supernatants as DNase-resistant EBV DNA. Cells were pelleted (300 × *g* for 10 min at 4°C), and supernatants were clarified (2,000 × *g* for 10 min at 4°C) and passed through 0.45-µm syringe filters to remove residual cells and debris. Clarified supernatants were aliquoted and subjected to DNase treatment or mock treatment in parallel. For DNase digestion, Turbo DNase I (Thermo Fisher) was added to a final concentration of 20 U/mL in the supplied buffer (final 5 mM MgCl_2_ and 1 mM CaCl_2_) and incubated 1 h at 37°C. Reactions were stopped by adding EDTA to 10 mM and heating to 75°C for 10 min, followed by lysis with proteinase K (200 µg/mL) and 0.5% SDS for 30 min at 55°C. Viral DNA was extracted from 200 to 500 µL of treated or mock-treated supernatant using the QIAamp MinElute Virus Spin Kit (Qiagen) and eluted in 50 µL of nuclease-free water. EBV DNA was quantified by qPCR targeting EBV genomic noncoding sequences using validated primer sets. Primer sequences are listed in Table S1. Absolute copy numbers were interpolated from plasmid standards.

### Infectious EBV production assay

GFP-EBV-positive Akata cells (Akata-EBV-eGFP) were treated with vehicle or IL-17A (500 ng/mL) to induce EBV reactivation. After 72 h, culture supernatants were collected and clarified by centrifugation to remove cells and debris. Supernatants were then filtered through a 0.45-μm membrane. Viral particles were concentrated from clarified supernatants by polyethylene glycol (PEG) precipitation according to the laboratory protocol. The resulting pellet was resuspended in RPMI medium and stored at −80°C until use. Concentrated viral preparations were added to Raji cells. After incubation, GFP-positive Raji cells were quantified by flow cytometry. Infectious EBV production was expressed as the percentage of GFP-positive Raji cells. Vehicle-treated donor cultures served as negative controls.

### RNA-seq analysis

For RNA-seq, Akata and Mutu I cells were treated with vehicle or IL-17A (500 ng/mL) for 48 h before RNA isolation. Total RNA was isolated from cells using the RNeasy Kit (Qiagen) with on-column DNase I treatment and assessed on an Agilent 2100 Bioanalyzer. Only samples with RIN ≥8 were sequenced. Strand-specific, poly(A)-enriched libraries were sequenced on a DNBSEQ platform (BGI) to generate paired-end 100-bp reads (PE100). Raw sequencing reads were aligned to a reference genome containing a human genome (hg38; Genome Reference Consortium GRCh38) plus a modified Akata-EBV genome (Akata—NCBI accession number KC207813.1 [28]). The alignments were conducted using the Spliced Transcripts Alignment to a Reference (STAR) aligner version 2.5.3 (--clip5pNbases 6, default options) (31) and were subjected to visual inspection using the Integrative Genomics Viewer (IGV) genome browser (32). Transcript data from STAR were then analyzed using the RSEM software (version 1.3.0 [33]) for quantification of human and EBV gene expression. Signal maps (i.e., the total number of reads covering each nucleotide position) were generated using the IGV tools, and read coverage maps were visualized using the IGV genome browser (32). The EBSeq software (34) was utilized to call statistically differentially expressed genes using a false discovery rate (FDR) <0.05. GSEA-Preranked was run on EBSeq Fold change/statistic (positive = up in IL-17A) using MSigDB Hallmark (v2025.1; symbols) with gene-set permutations (*n* = 10,000), min/max set sizes 15/500, and Collapse/Remap = No. Significance was assessed by NES and FDR *q*-value. To conduct GO:BP enrichment (pooled), a pooled IL-17A signature (Akata + Mutu) was generated from the combined differential expression analysis (one *P*-value per gene; consistent direction required). Overrepresentation testing used a one-tailed hypergeometric/Fisher’s exact test against GO:BP (Human), with Benjamini–Hochberg FDR correction where indicated; the gene universe was the union of expressed genes in either line. Dot plots display −log10(*P*) (or FDR) and gene ratio (overlap/term size).

### Statistical analysis

RT-qPCR, luciferase reporter assay, flow cytometry, and DNase-resistant viral DNA measurements were analyzed using GraphPad Prism. All statistical comparisons were performed using unpaired two-tailed Student’s *t*-test. Dose-response curves were fitted using a four-parameter logistic model to derive EC_50_ values with 95% confidence intervals. qPCR data were log_2_-transformed prior to statistical testing. All tests were two-sided, and data are presented as mean ± SEM unless otherwise stated. Statistical significance was defined as *P* < 0.05.

## RESULTS

### IL-17A induces EBV immediate-early transcription

**Fig 1:**
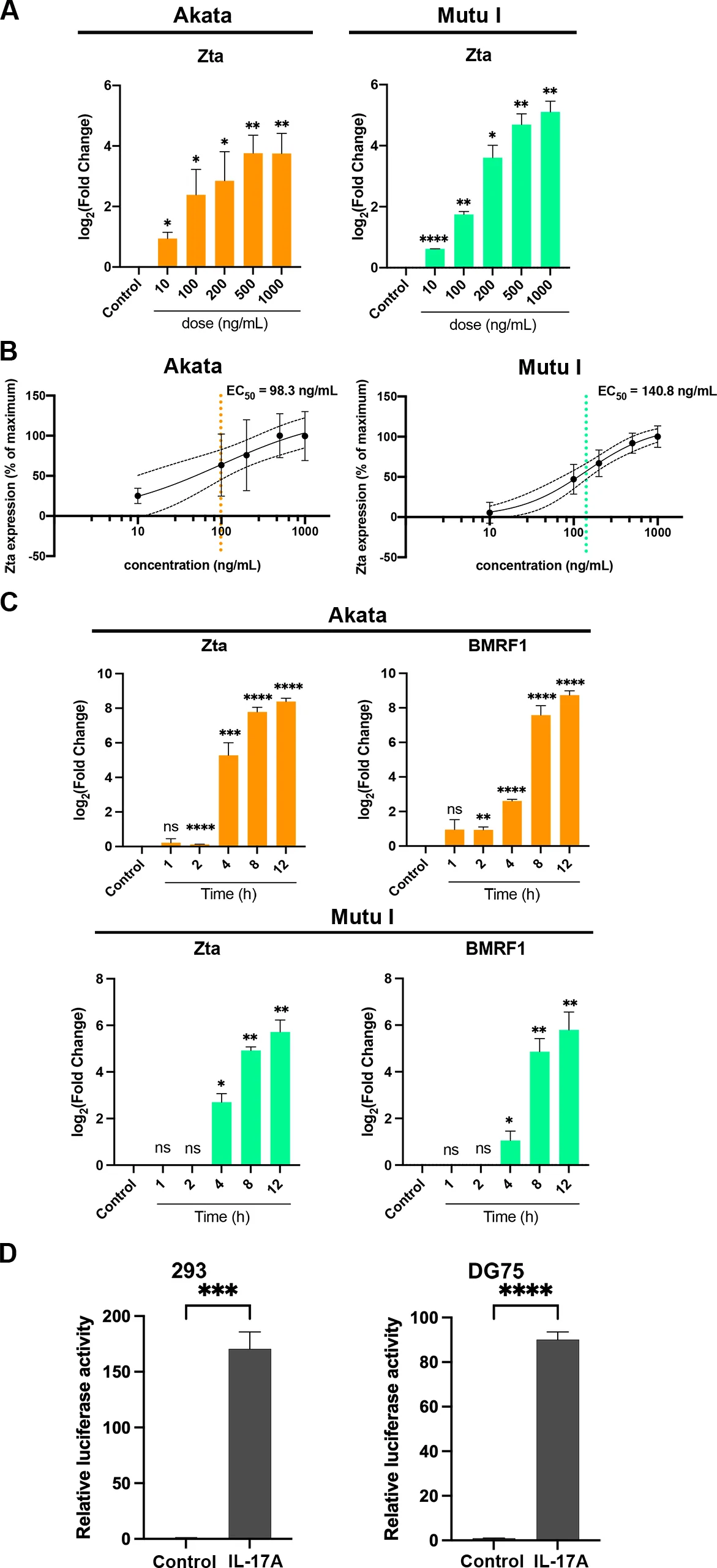
IL-17A induces EBV immediate-early transcription. (**A**) Akata and Mutu I cells were treated with increasing concentrations of IL-17A (10–1,000 ng/mL) for 48 h, and BZLF1 (Zta) mRNA was quantified by RT-qPCR. Expression was normalized to GAPDH and is shown as log_2_(fold change; 2^−ΔΔCt^) relative to untreated cells. (**B**) Dose-response curves for Zta induction in Akata and Mutu I cells. Fold changes from panel A were fit by nonlinear regression (four-parameter logistic), yielding EC_50_ values of 98.3 ng/mL (Akata) and 140.8 ng/mL (Mutu I). (**C**) Akata and Mutu I cells were treated with IL-17A (500 ng/mL) or control vehicle, and mRNA levels of BZLF1 (Zta) and BMRF1 were quantified by RT-qPCR at 1, 2, 4, 8, and 12 h. Zta/BZLF1 and BMRF1 were induced by 2 h in Akata cells and by 4 h in Mutu I cells, with both cell lines showing induction by 4 h, indicating rapid activation of immediate-early and early lytic transcription. (**D**) EBV-negative 293 and DG75 cells were transfected with a luciferase reporter driven by the BZLF1 promoter (Zp) and a Renilla control plasmid, then treated with control vehicle or IL-17A (500 ng/mL) for 24 h. Firefly activity was normalized to Renilla. For all RT-qPCR panels in this figure, bars/points represent means ± SEM from ≥2 biological replicates; each biological replicate was assayed in technical triplicate. Statistical significance was assessed by *t* test versus the corresponding control. ns, not significant; *, *P* < 0.05; **, *P* < 0.01; ***, *P* < 0.001; and ****, *P* < 0.0001.

To test whether IL-17A initiates EBV reactivation, EBV-positive Akata and Mutu I cells were exposed to recombinant IL-17A across 10–1,000 ng/mL, and BZLF1 (Zta) transcripts were quantified by RT-qPCR. Zta increased with dose, with statistically significant induction already observed at 10 ng/mL and maximal induction reached at approximately 500 ng/mL (Fig. 1A).

Nonlinear regression of the Zta dose response (four-parameter logistic on log_10_[IL-17A]) yielded an EC_50_ of 98.3 ng/mL (95% CI, 28.7–228.7; Hill slope = 0.63) in Akata cells and 140.8 ng/mL (95% CI, 98.6–190.0; Hill slope = 1.08) in Mutu I cells (Fig. 1B). Maximal induction was achieved at ≥500 ng/mL.

Thus, IL-17A responsiveness was detectable at lower *in vitro* concentrations, whereas 500 ng/mL was used for subsequent mechanistic experiments to define maximal, reproducible cytokine-driven reactivation. This concentration is supraphysiologic relative to circulating IL-17A levels, which are typically reported in the picograms per milliliter range (35, 36). For example, serum/plasma IL-17A values have been reported at approximately 1–55 pg/mL in inflammatory settings, with local inflammatory fluids sometimes higher (35, 36). Nevertheless, recombinant IL-17A is commonly used at nanograms per milliliter concentrations *in vitro* to elicit robust pathway activation in transformed or immortalized cell systems, where receptor abundance, cytokine stability, and assay sensitivity can limit responsiveness. Similar IL-17A concentration ranges (100–500 ng/mL) have been used to induce signaling in human keratinocytes, fibroblasts, endothelial/platelet systems, and immune-cell assays (37–43). Importantly, statistically significant Zta induction was already detectable at 10 ng/mL, and subinducing IL-17A concentrations enhanced BCR-mediated reactivation as described below.

To determine whether IL-17A activates EBV lytic transcription on a time scale compatible with cytokine receptor signaling, we performed an early RT-qPCR time-course experiment from 1 to 12 h after IL-17A treatment. Zta/BZLF1 transcripts were significantly induced by 2 h in Akata cells and by 4 h in Mutu I cells, with both cell lines showing induction by 4 h. BMRF1 transcripts also increased during this early time window (Fig. 1C). These data demonstrate rapid activation of immediate-early and early EBV lytic transcription after IL-17A treatment. A separate later time-course experiment from 16 to 72 h is also provided (Fig. S2) and shows dynamic regulation of Zta/BZLF1, BMRF1, and BcLF1/VCA during subsequent lytic progression. To determine whether IL-17A acts at the BZLF1 promoter (Zp), EBV-negative DG75 and 293 cells were transfected with a Zp-firefly luciferase reporter together with a Renilla luciferase control. IL-17A increased Renilla-normalized Zp-driven luciferase activity relative to vehicle, consistent with promoter-level activation (Fig. 1D).

### IL-17A drives full EBV lytic reactivation, DNase-resistant viral DNA release, and infectious virion production

**Fig 2:**
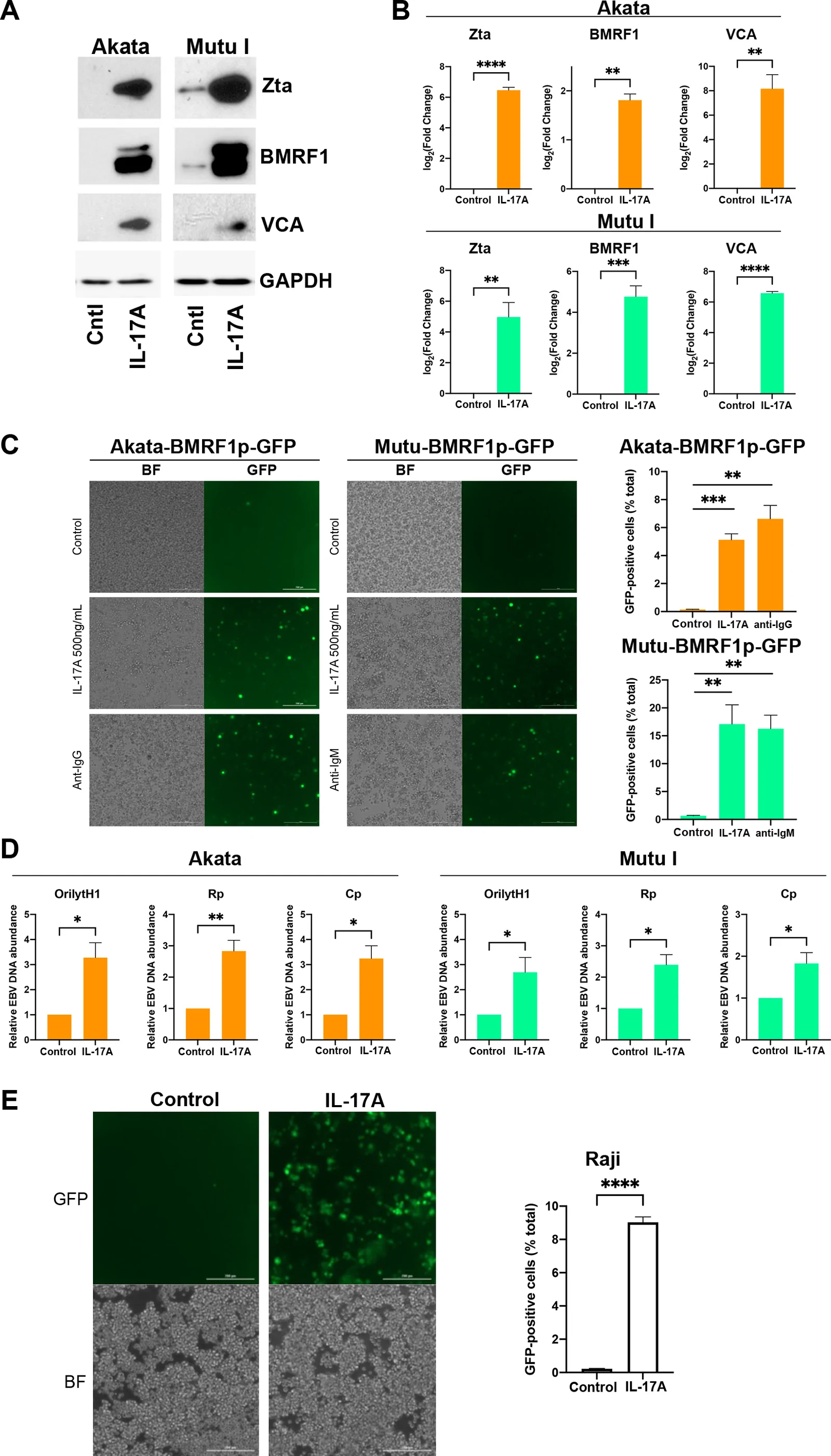
IL-17A drives full EBV lytic reactivation, DNase-resistant viral DNA release, and infectious virion production. (**A**) Akata and Mutu I cells were treated with control vehicle or IL-17A (500 ng/mL) for 48 h, and lysates were analyzed by immunoblotting with antibodies against Zta, BMRF1/EA-D, and p18/VCA. GAPDH served as a loading control. (**B**) RT-qPCR analysis of BZLF1, BMRF1, and BcLF1/VCA transcripts in Akata and Mutu I cells treated as in panel A. Expression was normalized to GAPDH and shown relative to vehicle-treated cells. (**C**) Akata and Mutu I cells stably expressing a lytic reporter (GFP under the BMRF1 promoter) were treated with control vehicle, IL-17A (500 ng/mL), or anti-Ig (10 µg/mL) for 48 h, and GFP-positive cells were quantified by flow cytometry. BF, bright field. (**D**) DNase-resistant, cell-free EBV DNA in culture supernatants from Akata and Mutu I cells treated with vehicle or IL-17A for 48 h was quantified by qPCR using primer pairs targeting distinct noncoding/non-transcribed regions of the EBV genome. (**E**) GFP-EBV-positive Akata cells were treated with control vehicle or IL-17A (500 ng/mL) for 72 h. Culture supernatants were collected, clarified, filtered through 0.45-µm filters, and subjected to PEG precipitation to concentrate EBV particles. PEG-concentrated viral preparations were then added to Raji cells. Infectious EBV production was quantified by flow cytometric measurement of GFP-positive Raji cells. For RT-qPCR and qPCR panels, data represent means ± SEM from ≥2 biological replicates; each biological replicate was assayed in technical triplicate. Statistical significance was assessed by *t* test versus the indicated control. *, *P* < 0.05; **, *P* < 0.01; ***, *P* < 0.001; and ****, *P* < 0.0001.

We next asked whether IL-17A advances beyond immediate-early induction to a full lytic program. EBV-positive Akata and Mutu I cells were treated with 500 ng/mL IL-17A for 48 h and analyzed by immunoblotting and RT-qPCR. IL-17A increased immediate-early (Zta), early (EA-D/BMRF1), and late (VCA/p18) proteins, with concordant induction of lytic transcripts (Fig. 2A and B).

An independent BMRF1 promoter-driven GFP reporter (10) further confirmed progression into the early lytic phase. Flow cytometric quantification showed that IL-17A increased the GFP-positive population to approximately 5.1% in Akata and 17.1% in Mutu I cells (Fig. 2C). As a benchmark for classical BCR-mediated reactivation, anti-Ig stimulation was tested in the same reporter system and induced approximately 6.1% GFP-positive Akata cells and 16.3% GFP-positive Mutu I cells. Thus, IL-17A induced early lytic reporter activation at levels comparable to anti-Ig stimulation under these assay conditions, supporting a biologically meaningful population-level entry into the lytic program.

To determine whether IL-17A promotes productive events, we quantified DNase-resistant, cell-free EBV DNA in culture supernatants. IL-17A significantly increased nuclease-protected viral DNA in both Akata and Mutu I supernatants (Fig. 2D), consistent with the presence of encapsidated viral genomes rather than naked DNA released from dying cells. To determine whether IL-17A-induced particles were infectious, we treated GFP-EBV-positive Akata cells with IL-17A, collected the culture supernatants, clarified them to remove cells and debris, filtered them through 0.45-µm filters, and concentrated viral particles by PEG precipitation. The resulting PEG-concentrated viral preparations were then added to Raji cells. Flow cytometric quantification showed that infection with viral preparations generated from IL-17A-treated cultures yielded significantly more GFP-positive Raji cells than preparations generated from vehicle-treated cultures (Fig. 2E). Together with the DNase-resistant extracellular EBV DNA data, these results indicate that IL-17A promotes productive reactivation culminating in infectious EBV particle production.

### IL-17A-induced lytic activation requires active cytokine, is sensitive to ganciclovir, and is selective among IL-17 family members

**Fig 3:**
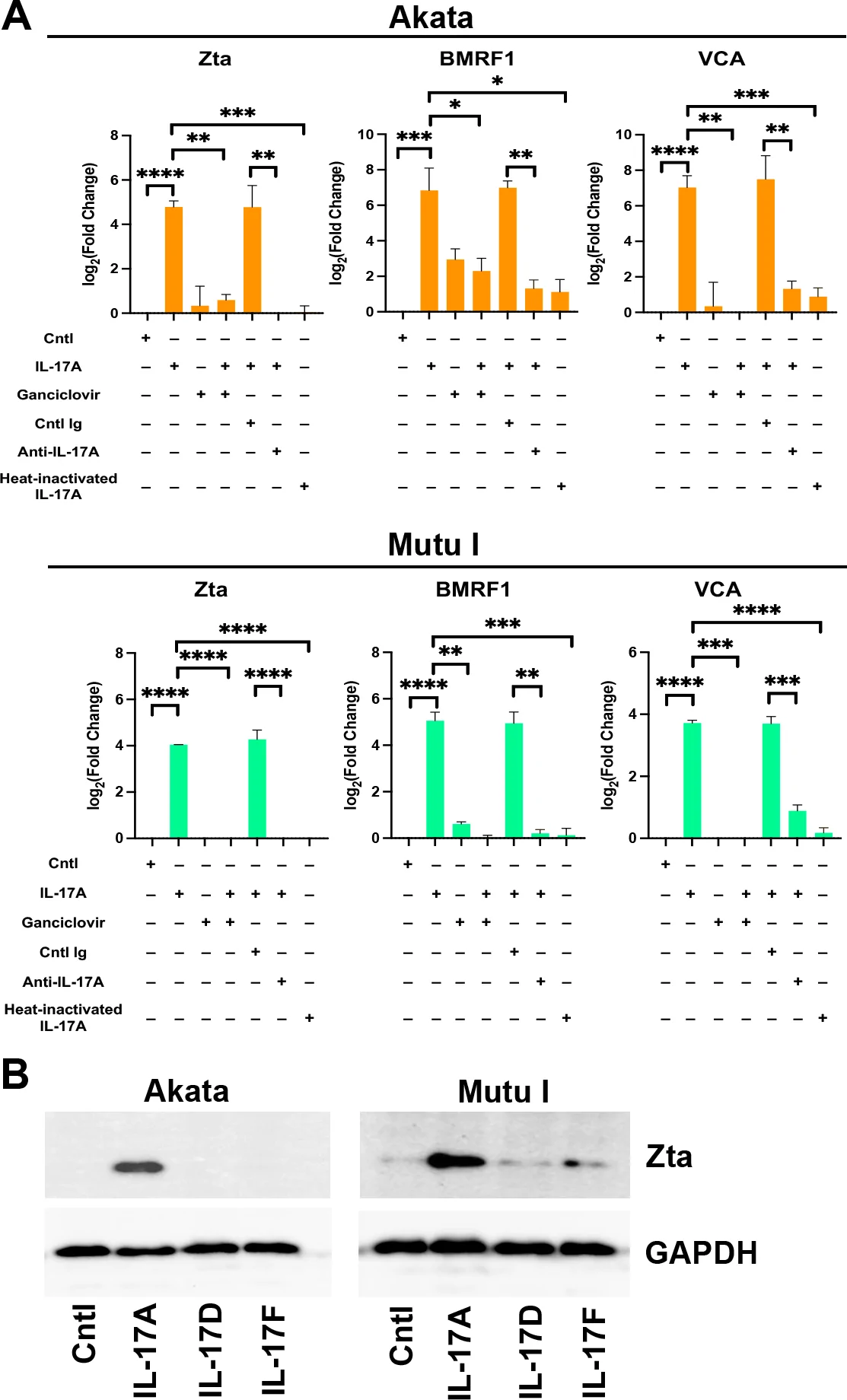
IL-17A-induced lytic transcription is ligand-dependent, sensitive to ganciclovir, and selective among IL-17 family members tested. (**A**) Akata and Mutu I cells were treated with control vehicle, IL-17A (500 ng/mL), IL-17A preincubated with neutralizing anti-IL-17A antibody, IL-17A preincubated with matched isotype control antibody (Cntl Ig), heat-inactivated IL-17A, ganciclovir, or IL-17A plus ganciclovir, as indicated for 24 h. EBV lytic transcripts were quantified by RT-qPCR. Neutralization and heat inactivation attenuated IL-17A-induced lytic transcript induction, whereas matched isotype control antibody did not. Ganciclovir reduced IL-17A-induced lytic transcript accumulation. For the early 4-h ganciclovir timing-control experiment, see Fig. S1. (**B**) Akata and Mutu I cells were treated with IL-17A, IL-17D, or IL-17F under matched conditions for 48 h. Only IL-17A induced detectable EBV lytic Zta activation. For RT-qPCR panels, expression was normalized to GAPDH and shown relative to control vehicle-treated cells. Data represent means ± SEM from biological replicates; each biological replicate was assayed in technical triplicate. Statistical significance was assessed by *t* test versus the indicated condition. *, *P* < 0.05; **, *P* < 0.01; ***, *P* < 0.001; and ****, *P* < 0.0001.

To establish ligand specificity and evaluate antiviral sensitivity, we compared IL-17A, IL-17A preincubated with neutralizing anti-IL-17A antibody, IL-17A preincubated with matched isotype control antibody, heat-inactivated IL-17A, ganciclovir, and IL-17A plus ganciclovir in the same experimental series. Neutralization and heat inactivation attenuated IL-17A-induced Zta/BMRF1/VCA transcript induction, indicating that active IL-17A is required and arguing against a nonspecific response to protein addition (Fig. 3A). Ganciclovir also reduced IL-17A-induced lytic transcript accumulation, consistent with suppression of replication-dependent lytic progression or amplification (Fig. 3A).

We next asked whether EBV reactivation was broadly induced by IL-17 family cytokines. Akata and Mutu I cells were treated with IL-17A, IL-17D, or IL-17F under matched conditions. IL-17A induced EBV immediate-early gene Zta expression, whereas IL-17D and IL-17F did not detectably induce reactivation (Fig. 3B). These data indicate that the response is selective for IL-17A among the IL-17 family members tested. Because ganciclovir reduced IL-17A-induced lytic transcript accumulation at the 24-h time point shown in Fig. 3A, we performed an early timing-control experiment to determine whether ganciclovir blocks the initial IL-17A-triggered lytic transcriptional response. At 4 h after IL-17A treatment, ganciclovir did not reduce IL-17A-induced Zta/BZLF1 or BMRF1 transcripts in Akata or Mutu I cells (Fig. S1). Thus, ganciclovir does not block the early IL-17A-induced immediate-early/early transcriptional response but reduces later lytic transcript accumulation at 24 h (Fig. 3A), consistent with inhibition of viral DNA replication-dependent lytic progression or amplification.

### IL-17A activates IL-17RA-linked NF-κB/MAPK signaling required for EBV reactivation

Having established that IL-17A induces productive EBV lytic reactivation, we next sought to define the proximal host signaling events that connect IL-17 receptor engagement to the viral lytic switch. IL-17 receptors canonically engage NF-κB and MAPK pathways, and these pathways are known regulators of EBV lytic promoter activity. We therefore directly examined whether IL-17A activates NF-κB/MAPK signaling in IL-17A-responsive latency I cells before evaluating transcriptome-wide responses.

**Fig 4:**
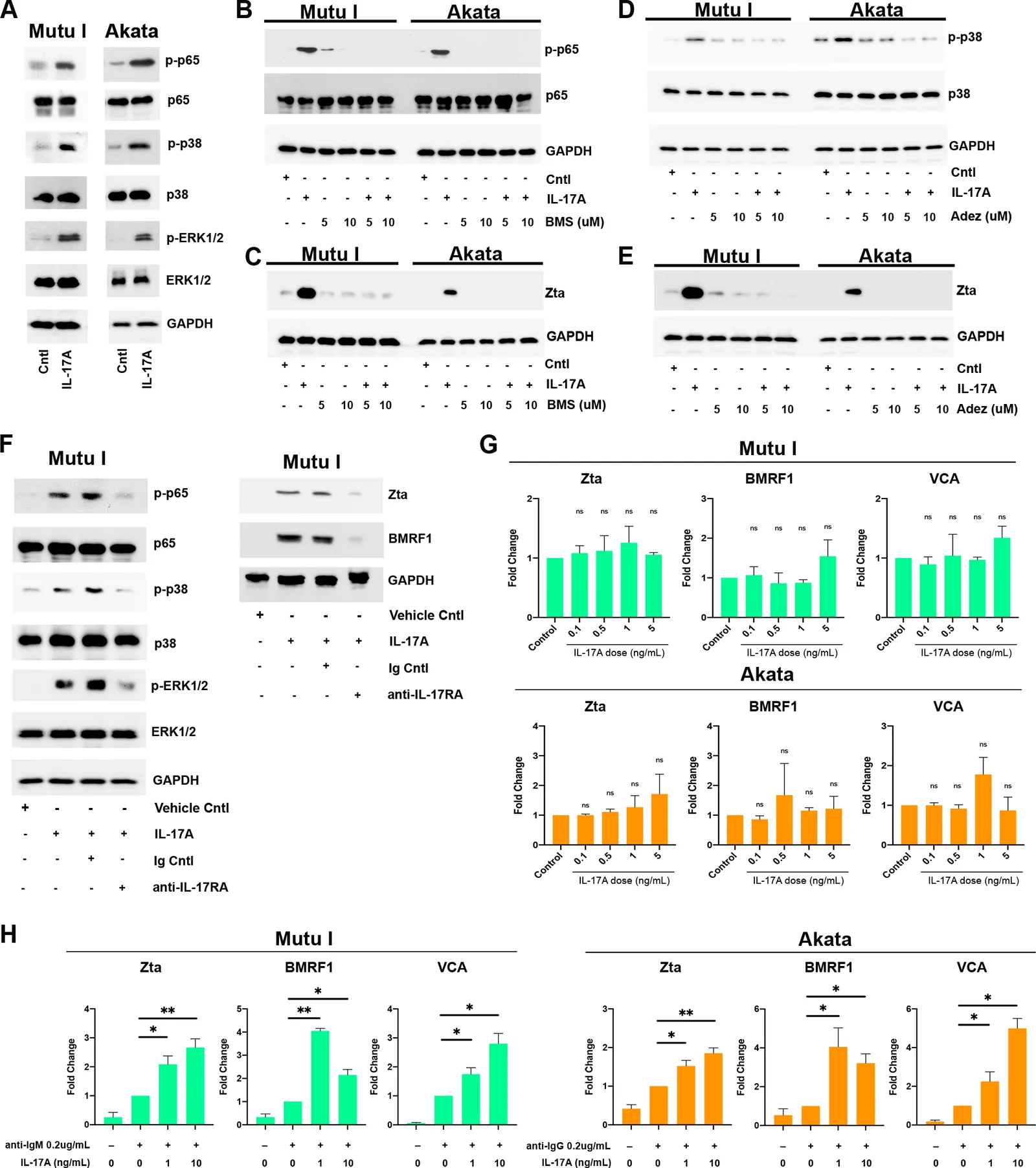
IL-17A activates IL-17RA-linked NF-κB/MAPK signaling required for EBV lytic reactivation. (**A**) Akata and Mutu I cells were treated with control vehicle or IL-17A (500 ng/mL) for 2 h, and whole-cell lysates were analyzed by immunoblotting for phospho-p65, phospho-ERK1/2, and phospho-p38. Total p65, ERK1/2, p38, and GAPDH served as controls. (**B–E**) Cells were pretreated with BMS-345541 (BMS) or Adezmapimod (Adez) for 1 h before IL-17A stimulation. For phosphorylation readouts, cells were then co-treated with IL-17A (500 ng/mL) and the indicated inhibitor for 2 h, and lysates were collected immediately after this 2-h stimulation. For Zta detection, cells were pretreated identically and co-treated with IL-17A plus inhibitor for 2 h. Inhibitor-containing medium was then replaced with fresh medium containing IL-17A or control vehicle, and cells were harvested 16 h after IL-17A addition. Inhibitor-alone controls were processed in parallel without IL-17A. BMS-345541 reduced IL-17A-induced p65 phosphorylation and Zta expression. Adezmapimod reduced IL-17A-associated p38 phosphorylation under our experimental conditions and attenuated Zta expression. (**F**) Mutu I cells were pretreated with anti-IL-17RA or matched isotype control for 30 min before IL-17A stimulation, and the antibody or isotype control was maintained in the culture medium throughout the subsequent IL-17A treatment. For phosphorylation detection, cells were harvested after 2 h of IL-17A stimulation. For lytic-marker detection, cells were harvested after 24 h of IL-17A stimulation. Phosphorylation of p65, ERK1/2, and p38, as well as EBV lytic protein expression, was assessed by immunoblotting. IL-17RA blockade reduced IL-17A-induced signaling and EBV lytic reactivation, whereas the matched isotype control did not. Immunoblots are representative of independent experiments. (**G and H**) Subinducing IL-17A (1 ng/mL) was insufficient to induce reactivation alone but enhanced subthreshold anti-Ig/BCR-mediated reactivation. Cells were treated with the indicated amount of IL-17A and/or anti-Ig for 24 h, and EBV lytic transcripts were quantified by RT-qPCR. Expression was normalized to GAPDH and shown as fold change relative to the indicated control. Data represent means ± SEM from biological replicates; each biological replicate was assayed in technical triplicate. ns, not significant; *, *P* < 0.05; **, *P* < 0.01; ***, *P* < 0.001; and ****, *P* < 0.0001.

Akata and Mutu I cells were treated with IL-17A, and early signaling was assessed by immunoblotting. IL-17A increased phosphorylation of NF-κB p65, ERK1/2, and p38, whereas total p65, ERK1/2, and p38 remained unchanged (Fig. 4A). These data indicate that IL-17A activates canonical NF-κB/MAPK signaling in latency I EBV-positive B cells.

To test whether early NF-κB and p38 MAPK signaling contributes to IL-17A-mediated EBV reactivation, cells were exposed to BMS-345541 or Adezmapimod during the early IL-17A signaling window. Inhibitor-alone controls showed Zta expression similar to vehicle control in the absence of IL-17A. BMS-345541 reduced IL-17A-induced p65 phosphorylation and attenuated Zta expression, while Adezmapimod reduced IL-17A-associated p38 phosphorylation under these experimental conditions and attenuated Zta expression (Fig. 4B through E). These results support functional involvement of NF-κB and p38 MAPK signaling in IL-17A-induced EBV lytic activation.

Next, we tested receptor dependence using IL-17RA blockade. Treatment with anti-IL-17RA reduced IL-17A-induced phosphorylation of p65, ERK1/2, and p38 and attenuated EBV lytic reactivation in Mutu I cells, whereas matched isotype control antibody did not block IL-17A-induced signaling or lytic protein expression (Fig. 4F). Together, these data support a receptor-linked signaling mechanism in which IL-17A activates NF-κB/MAPK pathways that are required for EBV lytic reactivation in latency I B cells.

To assess whether low IL-17A concentrations could contribute to EBV reactivation under co-stimulatory conditions, we first tested subthreshold IL-17A doses alone. IL-17A at 0.1, 0.5, and 1 ng/mL did not significantly induce lytic transcripts when applied alone. We then tested 1 ng/mL IL-17A in combination with subthreshold anti-Ig/BCR stimulation. Although 1 ng/mL IL-17A alone was inactive (Fig. 4G), it enhanced BCR-mediated reactivation under conditions in which BCR stimulation alone produced limited lytic induction (Fig. 4H). In contrast, 10 ng/mL IL-17A produced modest but statistically significant lytic transcript induction and further enhanced BCR-mediated reactivation (Fig. 1A and 4H). These data suggest that subinducing IL-17A can lower the threshold for antigen receptor-driven lytic entry.

### RNA-seq provides transcriptome-wide support for IL-17A-induced EBV lytic activation and host inflammatory programs

**Fig 5:**
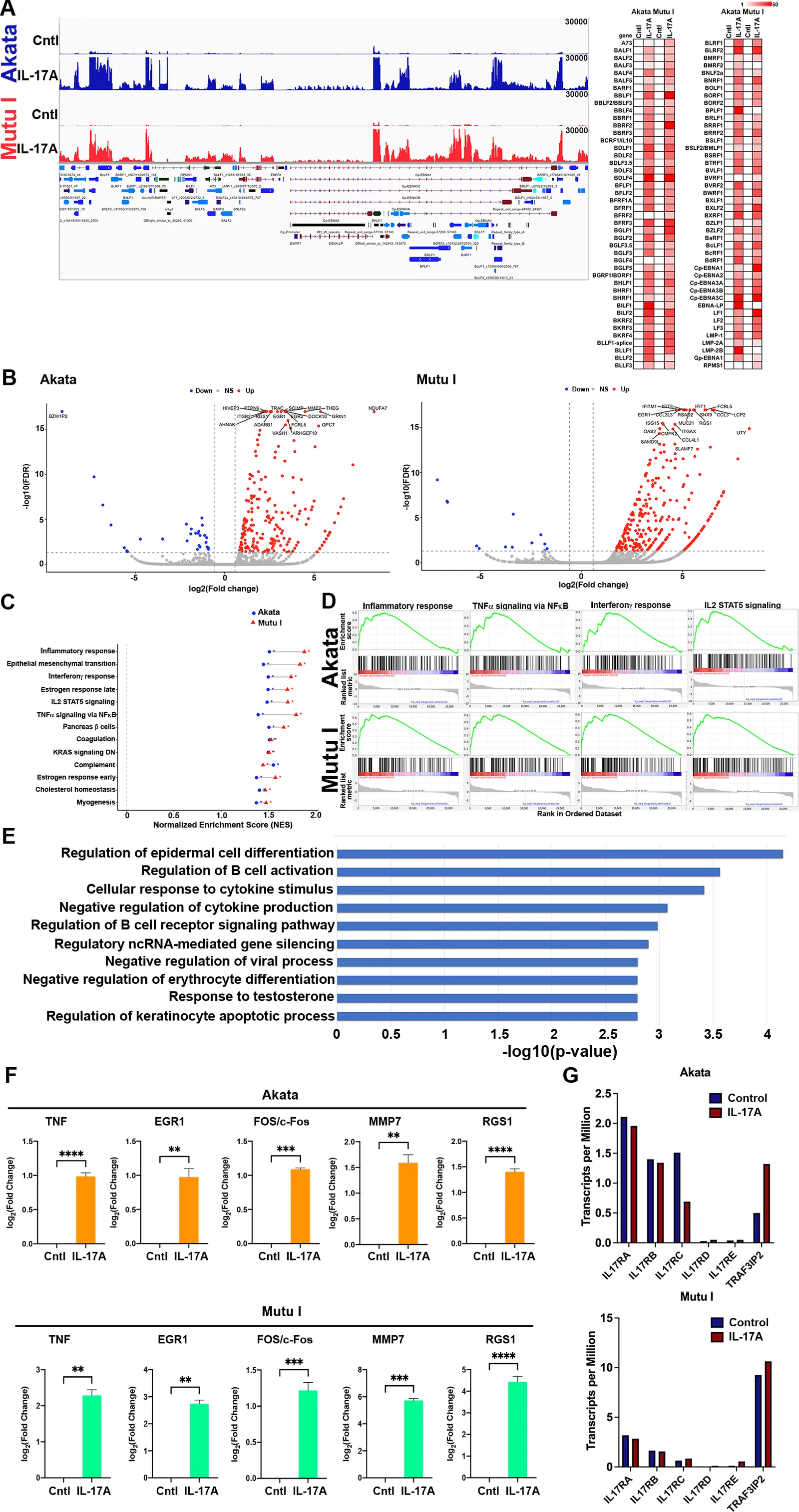
RNA-seq profiling provides transcriptome-wide support for IL-17A-induced EBV lytic activation and host inflammatory programs. (**A**) Akata and Mutu I cells were treated with control vehicle or IL-17A (500 ng/mL) for 48 h and subjected to DNBSEQ RNA-seq (paired-end 100 bp) to capture the overall viral and cellular response to IL-17A-mediated reactivation. Representative coverage across the EBV genome is shown using Integrative Genomics Viewer, together with a heat map of viral gene expression. (**B**) Volcano plots of host gene expression changes in Akata and Mutu I cells (IL-17A versus control). Dotted lines indicate thresholds (|log_2_ fold change| ≥ log_2_1.5; −log_10_FDR ≥ 1.3). Selected top altered genes are labeled. (**C and D**) Hallmark GSEA-Preranked identified 13 pathways significantly enriched (FDR *q* < 0.05) in both Akata and Mutu I cells after IL-17A treatment. Normalized enrichment scores for shared pathways are shown. (**E**) Pooled Gene Ontology Biological Process overrepresentation analysis of IL-17A-responsive genes from Akata and Mutu I highlights terms related to B-cell receptor signaling and B-cell activation. (**F**) RT-qPCR validation of representative host genes identified from RNA-seq/pathway analyses. Cells were treated with control vehicle or IL-17A (500 ng/mL) for 24 h. TNF, EGR1, FOS/c-Fos, MMP7, and RGS1 were selected to represent inflammatory/NF-κB signaling, MAPK/AP-1 immediate-early responses, IL-17A-responsive remodeling programs, and B-cell signaling regulation. Expression was normalized to GAPDH and shown as log_2_(fold change; 2^−ΔΔCt^) relative to control vehicle-treated cells. Data represent means ± SEM from biological replicates; each biological replicate was assayed in technical triplicate. Statistical significance was assessed by *t* test versus the corresponding control. *, *P* < 0.05; **, *P* < 0.01; ***, *P* < 0.001; and ****, *P* < 0.0001. (**G**) RNA-seq-based TPM values for IL-17 receptor subunits (IL17RA and IL17RC) and the adaptor TRAF3IP2 (ACT1) in Akata and Mutu I cells treated with control vehicle or IL-17A.

RNA-seq and pathway analyses are established approaches for identifying pathway-level transcriptional programs. To provide an orthogonal transcriptome-wide layer of evidence for IL-17A-mediated EBV reactivation, we performed RNA-seq on EBV-positive Akata and Mutu I cells treated with IL-17A or vehicle for 48 h. This time point was intentionally selected to capture the overall viral and cellular response after IL-17A treatment, including immediate-early, early, and late EBV lytic transcripts, rather than only proximal cytokine-receptor signaling. Reads were mapped to a combined human/EBV reference. Genome-wide read coverage across the EBV genome revealed broad increases in immediate-early (BZLF1/BRLF1), early (BMRF1), and late (BcLF1) lytic transcripts after IL-17A treatment (Fig. 5A). These viral transcriptome data are consistent with the RT-qPCR, immunoblotting, lytic reporter, DNase-resistant extracellular EBV DNA, and infectious-virus assays, supporting progression through the EBV lytic program. Host volcano plots highlighted the top 20 significantly altered cellular genes after IL-17A treatment, with most prominent changes corresponding to induced transcripts and fewer genes meeting the FDR threshold for repression (Fig. 5B).

To assess the alteration of host biological processes and signaling pathways, we next conducted the GSEA analysis using the human Hallmark gene sets as references. We identified 13 concordant pathways with FDR *q* < 0.05 in both Akata and Mutu I cell lines (Fig. 5C and D). Enriched sets were dominated by inflammatory modules, including inflammatory response, TNFα signaling via NF-κB, interferon gamma response, and IL2-STAT5 signaling with positive NES in Akata (1.37–1.55) and higher NES in Mutu (1.44–1.88). Additional concordant terms include epithelial-mesenchymal transition, complement, coagulation, cholesterol homeostasis, estrogen response early/late, myogenesis, pancreas beta cells, as well as KRAS signaling DN (i.e., genes downregulated by KRAS activation).

To increase functional resolution, we conducted an orthogonal GO Biological Process overrepresentation analysis on altered gene signatures after IL-17A treatment. The pooled GO analysis recovered B-cell-centric programs, including Regulation of B-cell receptor signaling pathway (GO:0050855; *P* ≈ 1.0 × 10^−3^), Regulation of B-cell activation (GO:0050864; *P* ≈ 2.7 × 10^−4^), and Cellular response to cytokine stimulus (GO:0071345; *P* ≈ 3.8 × 10^−4^), together with Negative regulation of cytokine production (GO:0001818; *P* ≈ 8.4×10^−4^), Negative regulation of viral process (GO:0048525; *P* ≈ 1.6 × 10^−3^), and Regulatory ncRNA-mediated gene silencing (GO:0031047; *P* ≈ 1.3 × 10^−3^) (Fig. 5E). These transcriptomic signatures are interpreted as the viral/host response accompanying IL-17A-mediated reactivation and are consistent with the biochemical data showing IL-17A-induced NF-κB/p38 MAPK activation. Thus, RNA-seq provides an additional transcriptome-wide layer of evidence, while proximal pathway involvement is supported by the phospho-signaling, IL-17RA blockade, isotype-control, and inhibitor experiments described above.

To validate representative host transcriptional changes identified by RNA-seq, we performed RT-qPCR for genes selected to span the major pathway signatures observed after IL-17A treatment, including inflammatory/NF-κB signaling (TNF), MAPK/AP-1 immediate-early responses (EGR1 and FOS/c-Fos), extracellular remodeling/IL-17A-responsive programs (MMP7), and B-cell signaling regulation (RGS1). IL-17A induced all five genes in both Akata and Mutu I cells (Fig. 5F), confirming the RNA-seq-defined inflammatory and signaling response at independent gene-level resolution. RNA-seq TPMs also documented the expression of IL17RA and IL17RC, the canonical IL-17 receptor subunits in both EBV-positive B-cell lines (Fig. 5G). IL17RA was readily detectable and minimally altered by IL-17A. IL17RC was lower in abundance and changed in opposite directions. Other paralogs (IL17RB/D/E) were low to very low and showed small absolute changes. The adaptor TRAF3IP2 (ACT1) was present in both lines, with an ~2.6-fold increase in Akata cells and an ~15% increase in Mutu I cells. Together, these data indicate that an IL-17RA-IL-17RC/ACT1 signaling axis is available in both models. These receptor/adaptor transcript data complement the functional evidence that IL-17A activates NF-κB/MAPK signaling and induces EBV lytic reactivation in latency I B-cell models.

### IL-17A responsiveness is restricted to latency I cells and correlates with inducible NF-κB/MAPK activation

**Fig 6:**
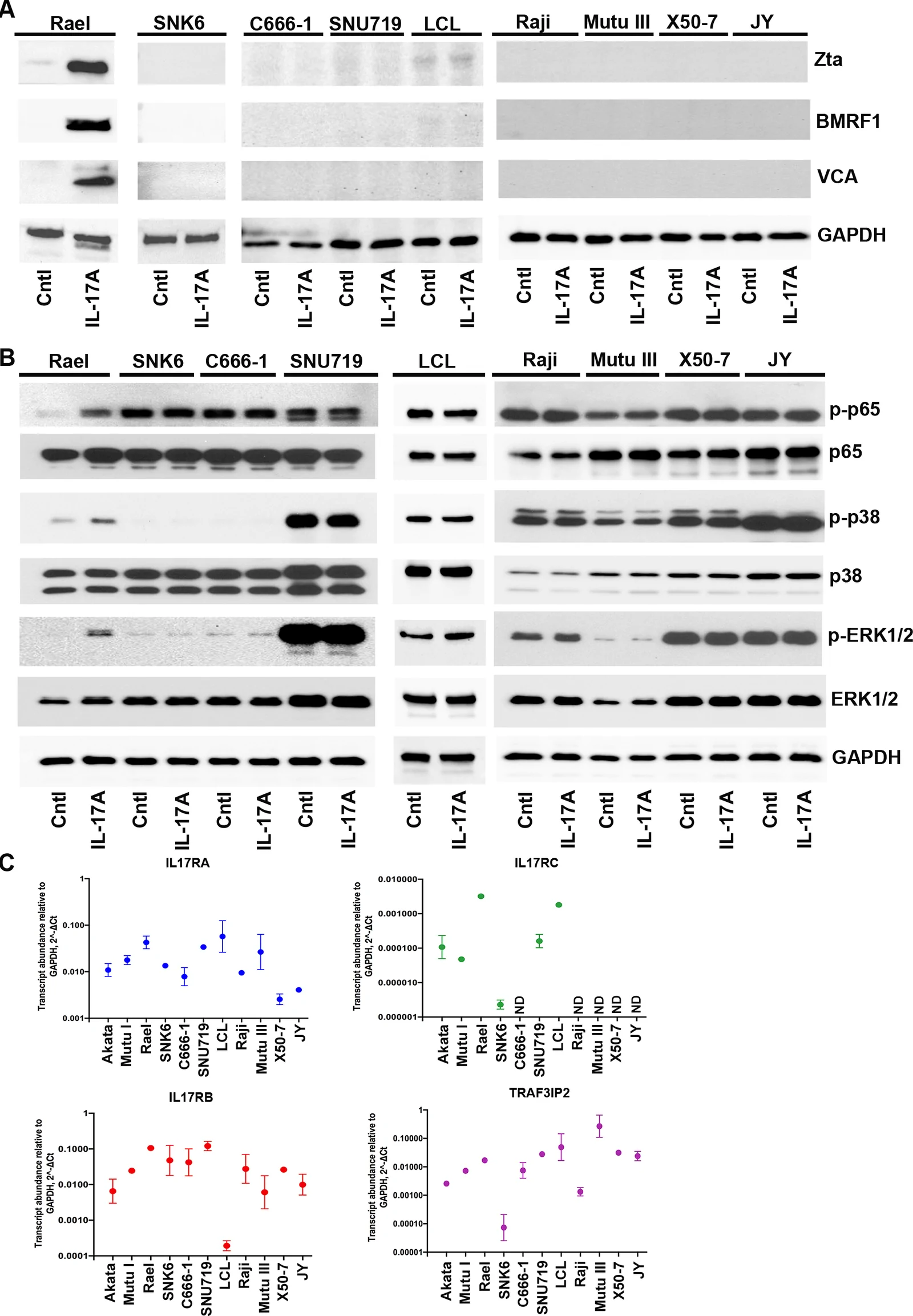
IL-17A responsiveness is latency- and cell-state restricted. (**A**) Rael (latency I), SNK6 (latency II), latency III B-cell models, LCL, and EBV-positive epithelial models were treated with control vehicle or IL-17A (500 ng/mL) for 48 h and analyzed for EBV lytic markers. IL-17A induced reactivation in latency I B-cell models but not in latency II/III, LCL, or epithelial contexts. (**B**) Cells were treated with control vehicle or IL-17A (500 ng/mL) for 2 h. IL-17A-induced signaling was assessed by immunoblotting for phosphorylated NF-κB p65, p38, and ERK1/2. Total p65, total p38, total ERK1/2, and GAPDH were included as controls for total protein abundance and loading. IL-17A induced p65, p38, and ERK1/2 phosphorylation in latency I-responsive cells (Rael) but did not elicit a robust coordinated increase in these phospho-signaling markers in latency II/III lymphocyte models or EBV-positive epithelial models. In LCLs, IL-17A produced at most a marginal change in ERK1/2 phosphorylation, without further increasing p65 or p38 phosphorylation above basal levels or inducing lytic protein expression. Several nonresponsive models exhibited basal phospho-NF-κB/MAPK signals, indicating that basal pathway activity alone is insufficient for EBV lytic induction. (**C**) IL-17 receptor family and TRAF3IP2/ACT1 transcript abundance across responsive and nonresponsive cell models was measured by RT-qPCR. Values are plotted as 2^−ΔCt^ relative to GAPDH, where ΔCt = Ct(target gene) − Ct(GAPDH). The *y*-axis is shown on a log10 scale. Higher values indicate higher transcript abundance. No cell-line calibrator was used for this baseline receptor-expression comparison. Each dot represents a biological replicate, with mean ± SEM shown where applicable. ND indicates no detectable amplification. IL17RD and IL17RE were not detected in the tested cell lines, whereas TRAF3IP2/ACT1 was detected in all tested cell lines.

Because Akata and Mutu I cells carry latency I, we next asked whether IL-17A could trigger reactivation in additional EBV latency programs and cell types. IL-17A induced lytic protein expression in the latency I Burkitt lymphoma line Rael, mirroring the responses observed in Akata and Mutu I (Fig. 6A). In contrast, IL-17A did not induce detectable lytic reactivation in latency II SNK6, latency III Raji, Mutu III, X50-7, JY, or human PBMC-derived LCL cells, nor in the EBV-positive epithelial models C666-1 and SNU719 (Fig. 6A).

To determine whether nonresponsiveness reflected a failure to activate IL-17A downstream signaling, we profiled NF-κB/MAPK pathway activation across this expanded cell panel. In the responsive latency I line Rael, IL-17A induced phosphorylation of NF-κB p65, p38, and ERK1/2, consistent with the signaling pattern observed in Akata and Mutu I cells (Fig. 6B). In contrast, IL-17A did not elicit a robust, coordinated increase in phospho-p65, phospho-p38, or phospho-ERK1/2 in the nonresponsive latency II, latency III/LCL, or epithelial models (Fig. 6B). In the PBMC-derived LCL model, any change in ERK1/2 phosphorylation was marginal and was not accompanied by increased p65 or p38 phosphorylation or by lytic protein induction. Several nonresponsive models nevertheless exhibited high basal pathway activity. For instance, untreated Raji, Mutu III, X50-7, JY, LCL, SNK6, C666-1, and SNU719 cells showed elevated basal phospho-p65. Untreated Raji, Mutu III, X50-7, JY, LCL, and SNU719 cells showed high basal phospho-p38. Untreated Raji, X50-7, JY, LCL, and SNU719 cells showed high basal phospho-ERK1/2.

These data indicate that IL-17A-induced EBV reactivation is not predicted simply by the presence of basal NF-κB/MAPK activity. Rather, productive coupling of IL-17A to EBV lytic entry requires inducible IL-17A-dependent NF-κB/MAPK activation in a latency I-permissive B-cell context. In nonresponsive latency II, latency III, LCL, and epithelial models, high basal signaling or lack of IL-17A inducibility is insufficient to activate the EBV lytic switch.

Finally, to determine whether nonresponsiveness reflected absence of IL-17 receptor-axis components, we quantified IL17RA, IL17RB, IL17RC, IL17RD, IL17RE, and TRAF3IP2/ACT1 transcripts across the cell panel by RT-qPCR. Because this analysis compared baseline transcript abundance across cell lines rather than treatment-induced fold change, values were plotted as 2^−ΔCt^ relative to GAPDH on a log_10_ scale, without using a cell-line calibrator (Fig. 6C). IL17RA, IL17RB, and/or IL17RC transcripts were variably detected across responsive and nonresponsive models, whereas IL17RD and IL17RE were not detected in the tested cell lines. In contrast, TRAF3IP2/ACT1 was detected in all tested cell lines. Together with the phospho-signaling data, these results indicate that receptor/adaptor transcript abundance alone does not predict IL-17A-induced EBV reactivation. Rather, reactivation requires inducible IL-17RA-linked NF-κB/MAPK signaling coupled to a latency I-permissive viral/cellular state.

## DISCUSSION

Our data show that interleukin-17A alone is sufficient to trigger EBV lytic reactivation and downstream productive events in latently infected human B cells. IL-17A induced rapid lytic transcription, with Zta/BZLF1 and BMRF1 transcripts increasing as early as 2–4 h, followed by broader early/late lytic transcription, lytic protein expression, and productive output. Importantly, culture supernatants from IL-17A-treated cells contained significantly higher levels of DNase-resistant extracellular EBV DNA, consistent with nuclease-protected viral genomes. We further show that PEG-concentrated viral preparations generated from filtered supernatants of IL-17A-treated GFP-EBV Akata cultures infected Raji cells and produced significantly increased GFP-positive Raji cells, demonstrating production of infectious EBV particles. These findings indicate that IL-17A not only initiates lytic entry but also supports productive stages culminating in infectious virion release.

The new mechanistic data support a receptor-linked NF-κB/MAPK model for IL-17A-driven EBV reactivation. IL-17A increased phosphorylation of p65, ERK1/2, and p38 in latency I Akata and Mutu I cells, while total protein levels remained stable. IL-17RA blockade reduced these phosphorylation events and attenuated reactivation, whereas matched isotype control antibody did not block IL-17A-induced signaling or lytic protein expression. Moreover, NF-κB and p38 inhibitors reduced IL-17A-induced p65 and p38 phosphorylation and suppressed EBV lytic readout, establishing NF-κB and p38 MAPK as a functional requirement for IL-17A-mediated reactivation. These data move the model beyond transcriptomic inference and show that IL-17A activates host signaling pathways directly linked to the EBV lytic switch.

The expanded latency II, latency III, LCL, and epithelial cell analyses refine this model. IL-17A did not induce EBV reactivation in SNK6, Raji, Mutu III, X50-7, JY, PBMC-derived LCL, C666-1, or SNU719, demonstrating that IL-17A does not act as a generic stressor capable of triggering lytic replication in all EBV-positive cells. Importantly, IL-17A also failed to elicit a robust, coordinated increase in phospho-p65, phospho-p38, or phospho-ERK1/2 in the nonresponsive models tested, whereas the latency I line Rael showed inducible phosphorylation of all three markers, similar to Akata and Mutu I. In LCLs, any ERK1/2 phosphorylation change was marginal and was not accompanied by an IL-17A-induced increase in p65 or p38 phosphorylation above basal levels or by EBV lytic protein induction. This indicates that isolated or weak ERK activation is insufficient to trigger reactivation in latency III/LCL contexts. Several nonresponsive cells nevertheless displayed high basal pathway phosphorylation, indicating that basal NF-κB/MAPK activity alone is not sufficient to activate the EBV lytic switch. Receptor/adaptor profiling further showed variable IL17RA/IL17RB/IL17RC transcript abundance across the panel, whereas IL17RD and IL17RE were not detected. TRAF3IP2/ACT1 was detected in all tested cell lines, indicating that absence of ACT1 transcript does not explain nonresponsiveness. These findings suggest that IL-17A-driven reactivation requires two coupled conditions: inducible IL-17RA-linked NF-κB/MAPK activation and a latency I viral/cellular state permissive for Zp/Rp activation. In latency II, latency III, LCL, or epithelial contexts, tonic pathway activation, lack of IL-17A inducibility, latency-associated transcriptional programs, viral chromatin state, or cofactor availability may uncouple inflammatory signaling from lytic promoter activation.

Our findings also align with emerging work showing that cytokines can regulate EBV transcriptional programs in B cells. Liao et al. (18) recently showed that germinal center cytokines, including IL-2 and IL-15, reshape EBV latency gene expression through JAK/STAT-dependent epigenetic control and can induce lytic-associated proteins in LCL contexts. In contrast, IL-17A did not reactivate the LCL/type III models tested here but did trigger reactivation in latency I B-cell models, highlighting cytokine- and latency-program-specific routes by which inflammatory signals influence EBV gene expression.

The dose-response experiments establish that IL-17A can act as a sufficient cytokine trigger of EBV reactivation when present at concentrations that produce robust pathway activation *in vitro*. Importantly, significant Zta induction was detectable at 10 ng/mL, indicating that IL-17A responsiveness is not limited to the 500 ng/mL dose used for maximal and reproducible mechanistic studies. We recognize that 500 ng/mL exceeds typical circulating IL-17A levels, which are generally in the picograms per milliliter range. However, local cytokine exposure in inflamed tissue niches may differ from plasma measurements, and 10–500 ng/mL IL-17A is widely used for *in vitro* pathway activation assays. Our subinducing IL-17A co-stimulation data refine this interpretation. IL-17A at 0.5–1 ng/mL was insufficient to induce detectable reactivation alone, but 1 ng/mL IL-17A enhanced subthreshold BCR-mediated reactivation. Thus, high-dose IL-17A demonstrates sufficiency, 10 ng/mL demonstrates lower-dose responsiveness, and subinducing IL-17A concentrations may function physiologically as threshold-lowering inflammatory co-signals in B cells receiving antigen receptor stimulation.

Having established the dose-dependent and context-dependent nature of the response, we next considered whether IL-17A acts through active cytokine signaling rather than nonspecific protein addition or generalized stress. Specificity is supported by ligand neutralization and heat inactivation, both of which attenuated IL-17A-induced lytic transcript induction, whereas matched isotype control antibody did not. The ganciclovir timing-control experiments further clarified the role of viral DNA replication in this response. Ganciclovir did not reduce early IL-17A-induced Zta/BZLF1 or BMRF1 transcripts at 4 h, indicating that initial immediate-early/early induction does not require viral DNA replication. At later time points, ganciclovir reduced accumulation of Zta, BMRF1, and BcLF1/VCA transcripts, supporting a replication-dependent component of lytic progression and amplification. Most importantly, IL-17RA blockade reduced IL-17A-induced phosphorylation of p65, ERK1/2, and p38 and attenuated EBV lytic reactivation. NF-κB/p38 MAPK pathway inhibition suppressed IL-17A-induced p65/p38 phosphorylation and reactivation. These findings support a receptor-linked NF-κB/MAPK mechanism rather than nonspecific stress signaling.

Ligand comparison further supports IL-17A specificity. Among the IL-17 family members tested under matched conditions, IL-17D and IL-17F did not detectably induce EBV immediate-early gene Zta expression, whereas IL-17A did. These results indicate that EBV lytic induction is not a general response to IL-17 family cytokines. Although IL-17F can share receptor usage with IL-17A in some settings, its failure to induce reactivation here suggests that IL-17A-specific ligand potency, receptor engagement, or signaling amplitude is required to couple IL-17 receptor signaling to the EBV lytic switch in latency I B cells.

Classically, EBV lytic reactivation in B cells has been modeled using B-cell receptor cross-linking, phorbol esters/HDAC inhibitors, or tissue-level cues such as hypoxia/HIF-1α, COX-2/PGE2, and TGF-β. These stimuli converge on Zp/Rp via AP-1, CREB/ATF, NF-κB, and chromatin remodeling. By contrast, IL-17A is a defined immune cytokine present in inflamed niches. The demonstration that IL-17A alone elicits both lytic entry and DNase-resistant EBV DNA elevates cytokine signaling from a permissive inflammatory backdrop to a sufficient upstream trigger of productive reactivation in latency I B-cell contexts.

Our transcriptome analyses provide an additional layer of support for the biochemical model and a new genome-wide view of the viral and host response to IL-17A in human B-cell models. The 48-h RNA-seq time point was selected to monitor the overall response to IL-17A-mediated reactivation, including immediate-early, early, and late viral transcription, which would not be fully captured by very early transcriptome profiling. Line-specific Hallmark GSEA identified 13 concordant pathways dominated by inflammatory response, TNFα signaling via NF-κB, interferon-γ response, and IL-2/STAT5 signaling. Pooled GO:BP analysis recovered regulation of B-cell receptor signaling, regulation of B-cell activation, and cellular response to cytokine stimulus. RT-qPCR validation confirmed IL-17A-dependent induction of representative host genes. These RNA-seq and pathway data are used here as an orthogonal transcriptome-wide readout of the viral and host programs accompanying IL-17A-mediated reactivation, while the proximal mechanism is defined by early phospho-signaling, IL-17RA blockade, isotype-control experiments, and NF-κB/p38 inhibitor studies.

**Fig 7:**
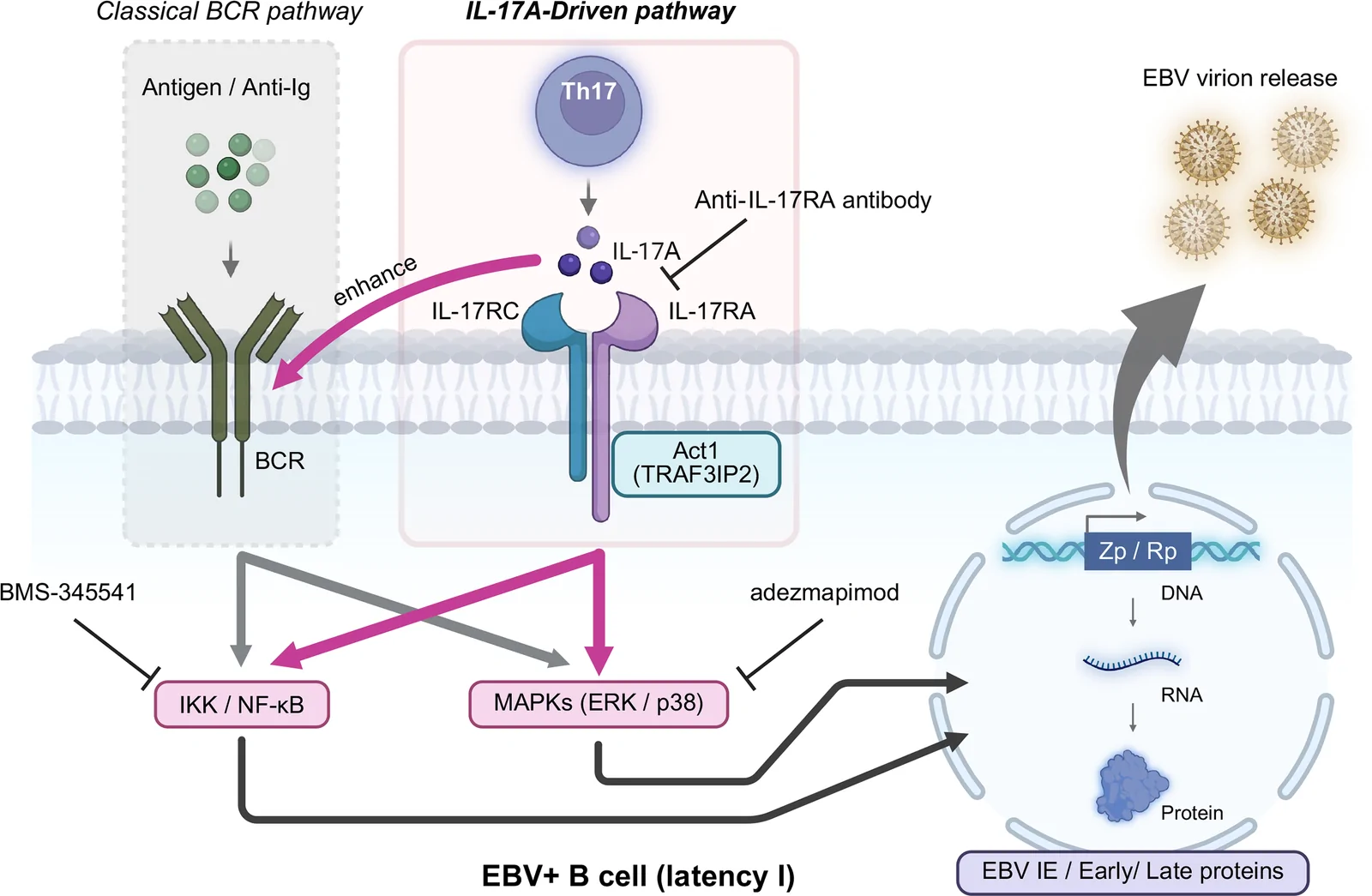
Model of latency-restricted IL-17A-mediated EBV lytic reactivation. In latency I EBV-positive B cells, IL-17A engages IL-17RA/IL-17RC and Act1/TRAF3IP2 to induce NF-κB/MAPK signaling, including phosphorylation of p65, p38, and ERK1/2. IL-17RA blockade reduces these signaling events and attenuates EBV lytic reactivation, and NF-κB or p38 MAPK inhibition suppresses IL-17A-induced p65 or p38 phosphorylation and reactivation. In this permissive latency I context, inducible IL-17A signaling couples to Zp/Rp activation, BZLF1/BRLF1 induction, downstream early/late lytic gene expression, and infectious virion production. In contrast, latency II, latency III, LCL, and EBV-positive epithelial models fail to undergo IL-17A-induced reactivation. Several nonresponsive cells show high basal p65, p38, or ERK1/2 phosphorylation, but IL-17A does not elicit a robust coordinated increase in these pathways. These data support a model in which basal NF-κB/MAPK activity is insufficient. IL-17A-driven EBV reactivation requires inducible IL-17R-linked signaling and a latency I-permissive viral/cellular state. Created in BioRender. Dochi, H. (2026) https://BioRender.com/hfm9qcv.

Our findings also clarify the relationship between IL-17A and classical BCR-mediated EBV reactivation (Fig. 7). BCR engagement activates proximal SYK/BTK-PLCγ2-PKCβ signaling and the CARD11-BCL10-MALT1 complex, whereas IL-17A signals through IL-17RA/IL-17RC and Act1/TRAF3IP2 (19, 44–49). These are distinct upstream pathways, and our data do not imply that IL-17A binds to BCR or requires BCR co-stimulation. Rather, both inputs can feed into overlapping downstream NF-κB/MAPK outputs that are relevant to Zp/Rp activation (14, 17, 50). In support of this model, IL-17A induced p65, ERK1/2, and p38 phosphorylation. IL-17RA blockade reduced these signaling events and lytic reactivation, and NF-κB/p38 MAPK inhibition suppressed IL-17A-induced p65/p38 phosphorylation and EBV reactivation. In addition, subinducing IL-17A was insufficient to reactivate EBV alone but enhanced subthreshold BCR-mediated reactivation, suggesting that IL-17A can lower the threshold for antigen receptor-driven lytic entry in inflammatory B-cell niches. However, convergence on NF-κB/MAPK outputs is productive only in a permissive cellular context. Nonresponsive latency II, latency III/LCL, and epithelial models showed no IL-17A-induced robust coordinated increase in p65, p38, or ERK1/2 phosphorylation, and several displayed high basal phosphorylation without lytic induction. Thus, IL-17A and BCR are best viewed as parallel upstream inputs that converge functionally on NF-κB/MAPK-dependent activation of the EBV lytic switch in latency I-permissive B cells. Canonical TRAF6/TAK1 intermediates may participate in this convergence, but direct testing of TAK1 remains an important future direction.

These findings have pathobiologic and translational implications. Many EBV-associated settings display Th17-skewed inflammation with elevated IL-17A. Our results suggest that IL-17A-rich microenvironments may promote episodic, productive reactivation or lower the threshold for reactivation in B cells receiving additional signals, increasing local virion burden and antigen exposure. A feed-forward model is therefore plausible. That is, EBV products can stimulate IL-17A responses, and IL-17A can reciprocally augment EBV reactivation, establishing an inflammation-reactivation loop. Because we detect DNase-resistant extracellular EBV DNA after IL-17A exposure, future studies could correlate IL-17A-rich tissue environments with cell-free EBV DNA or other reactivation markers *in vivo*.

### Conclusions

Our data identify IL-17A as a receptor-linked cytokine trigger of EBV lytic reactivation in latency I human B cells. The response is supported by rapid induction of EBV lytic transcripts, direct activation of NF-κB/MAPK signaling, and reduction by IL-17RA blockade and NF-κB/MAPK inhibition. IL-17A responsiveness is latency- and cell-state restricted, indicating that receptor signaling must be coupled to a permissive EBV lytic switch. Subinducing IL-17A (1 ng/mL) enhances BCR-mediated reactivation, suggesting that IL-17A can function as a threshold-lowering inflammatory cue in B-cell niches. Together, these findings place IL-17A signaling alongside established EBV lytic inducers and provide a mechanistic framework for understanding how Th17-skewed inflammation shapes EBV reactivation burden.

## Data Availability

The RNA-seq data generated in this study have been deposited in the NIH Sequence Read Archive (SRA) under BioProject accession number PRJNA1390600. All other data supporting the findings of this study are included in the main text and supplemental material. RNA-seq analyses used publicly available software and databases, including STAR (https://github.com/alexdobin/STAR), RSEM (https://deweylab.github.io/RSEM/), EBSeq (https://bioconductor.org/packages/EBSeq/), IGV (https://igv.org/), GSEA (https://www.gsea-msigdb.org/gsea/index.jsp), and MSigDB (https://www.gsea-msigdb.org/gsea/msigdb).

## SUPPLEMENTAL MATERIAL

Fig. S1 jvi.00813-26-s0001.tif Ganciclovir does not block early IL-17A-induced Zta/BZLF1 or BMRF1 transcription.

Fig. S2 jvi.00813-26-s0002.tif Later time-course analysis of IL-17A-induced EBV lytic transcripts.

Supplemental legends jvi.00813-26-s0003.docx Descriptive legends for Fig. S1 and S2.

Table S1 jvi.00813-26-s0004.xlsx Oligonucleotide primers used for RT-qPCR and EBV DNA qPCR assays.

ASM does not own the copyrights to Supplemental Material that may be linked to, or accessed through, an article. The authors have granted ASM a non-exclusive, world-wide license to publish the Supplemental Material files. Please contact the corresponding author directly for reuse.
