# Supplementary material for: Interleukin-17A directly triggers Epstein-Barr virus lytic reactivation in latency I human B-cell models
Source: J Virol. 2026 Sep 24;100(10):e00813-26. doi: 10.1128/jvi.00813-26 (PMC13712830; doi:10.1128/jvi.00813-26)
Supplement: Supplementary file 1 [file jvi.00813-26-s0003.docx]

**SUPPLEMENTAL MATERIAL**

**FIG S1. Ganciclovir does not block early IL-17A-induced Zta/BZLF1 or BMRF1 transcription.**
Akata and Mutu I cells were treated with control vehicle, IL-17A (500 ng/mL), ganciclovir, or IL-17A plus ganciclovir for 4 h. Zta/BZLF1 and BMRF1 transcripts were quantified by RT-qPCR. Ganciclovir did not reduce early IL-17A-induced Zta/BZLF1 or BMRF1 transcript induction. Data represent means ± SEM from biological replicates. Each biological replicate was assayed in technical triplicate. ns, not significant; *, P < 0.05; **, P < 0.01; ***, P < 0.001; ****, P < 0.0001. For the 24-h effect of ganciclovir on lytic transcript accumulation, see Fig. 3A.

**FIG S2. Later time-course analysis of IL-17A-induced EBV lytic transcripts.**
Akata and Mutu I cells were treated with IL-17A (500 ng/mL) or control vehicle, and Zta/BZLF1, BMRF1, and BcLF1/VCA transcripts were quantified by RT-qPCR at later time points from 16 to 72 h. Expression was normalized to GAPDH and is shown as log2(fold change; 2^-ΔΔCt) relative to time-matched controls. Data represent means ± SEM from biological replicates; each biological replicate was assayed in technical triplicate. ns, not significant; *, P < 0.05; **, P < 0.01; ***, P < 0.001; ****, P < 0.0001.
